# MEG reveals a fast pathway from somatosensory cortex to occipital areas via posterior parietal cortex in a blind subject

**DOI:** 10.3389/fnhum.2013.00429

**Published:** 2013-08-05

**Authors:** Andreas A. Ioannides, Lichan Liu, Vahe Poghosyan, George A. Saridis, Albert Gjedde, Maurice Ptito, Ron Kupers

**Affiliations:** ^1^Laboratory for Human Brain Dynamics, AAI Scientific Cultural Services Ltd.Nicosia, Cyprus; ^2^BRAINlab, Department of Neuroscience and Pharmacology, Faculty of Health and Medical Sciences, University of CopenhagenCopenhagen, Denmark; ^3^Harland Sanders Chair, School of Optometry, University of MontrealMontreal, QC, Canada

**Keywords:** cortico-cortical connections, brain plasticity, cross-modal perception, cross-modal plasticity, functional connectivity, time-delayed mutual information, MEG tomography

## Abstract

Cross-modal activity in visual cortex of blind subjects has been reported during performance of variety of non-visual tasks. A key unanswered question is through which pathways non-visual inputs are funneled to the visual cortex. Here we used tomographic analysis of single trial magnetoencephalography (MEG) data recorded from one congenitally blind and two sighted subjects after stimulation of the left and right median nerves at three intensities: below sensory threshold, above sensory threshold and above motor threshold; the last sufficient to produce thumb twitching. We identified reproducible brain responses in the primary somatosensory (S1) and motor (M1) cortices at around 20 ms post-stimulus, which were very similar in sighted and blind subjects. Time-frequency analysis revealed strong 45–70 Hz activity at latencies of 20–50 ms in S1 and M1, and posterior parietal cortex Brodmann areas (BA) 7 and 40, which compared to lower frequencies, were substantially more pronounced in the blind than the sighted subjects. Critically, at frequencies from α-band up to 100 Hz we found clear, strong, and widespread responses in the visual cortex of the blind subject, which increased with the intensity of the somatosensory stimuli. Time-delayed mutual information (MI) revealed that in blind subject the stimulus information is funneled from the early somatosensory to visual cortex through posterior parietal BA 7 and 40, projecting first to visual areas V5 and V3, and eventually V1. The flow of information through this pathway occurred in stages characterized by convergence of activations into specific cortical regions. In sighted subjects, no linked activity was found that led from the somatosensory to the visual cortex through any of the studied brain regions. These results provide the first evidence from MEG that in blind subjects, tactile information is routed from primary somatosensory to occipital cortex via the posterior parietal cortex.

## Introduction

Blindness improves performance in a variety of non-visual tasks (Lessard et al., 1998; Kupers et al., 2010; Kupers and Ptito, 2011). Positron emission tomography (PET) and functional magnetic resonance imaging (fMRI) studies show that such improvements are associated with activity in visual cortex during the performance of auditory (Kujala et al., 1995), somatosensory (Sadato et al., 1996) or cognitive tasks (Kupers et al., 2010). In addition to responses in visual cortex (Buchel et al., 1998; Burton et al., 2002; Kupers et al., 2010), studies in congenitally blind subjects have identified cross-modal responses also in the posterior parietal cortex, more specifically in Brodmann areas (BA) 7 and 40. These dorsal parietal areas, as well as sub-cortical structures, are perfectly suited as intermediate conduits for non-visual inputs to the visual cortex. Thus, they could contribute to the excitation of visual cortex observed in congenitally blind subjects (Ptito et al., 2005). The key questions remain through which pathways and at what latencies non-visual inputs are funneled to the visual cortex.

In the present study, we provide a detailed analysis of the spatio-temporal profile of brain responses to simple non-visual stimuli in a congenitally blind participant who has consistently demonstrated activation of the visual cortex in response to a wide variety of non-visual stimuli (Ptito et al., 2005, 2012; Kupers et al., 2010; Matteau et al., 2010; Kupers and Ptito, 2011). We also compare these activations with those obtained in sighted subjects.

We hypothesize that simple median nerve stimulation will activate the visual cortex of blind subjects. We further hypothesize that the magnitude of activity in key brain regions will increase with the intensity of the stimulus. By comparing brain responses to varying stimulus intensities we largely eliminate the effects of strategy and other subjective factors and therefore can safely draw firm conclusions at the level of an individual subject. By studying in detail the time-dependent connections between selected brain regions we can trace the putative pathway from the somatosensory to the visual cortex.

The specific goals of our data analysis are to identify (1) the early stimulus-evoked responses in the primary somatosensory (S1) and motor (M1) cortices in the blind subject and determine if such responses differ from sighted subjects; (2) the characteristics of these responses in the time and frequency domain; (3) strong changes in activity in the visual cortex of the blind subject that increase with the intensity of the stimulus; and (4) to determine if somatosensory stimulation in sighted subjects leads to activations in the visual cortex and if so, how they differ from the blind subject. Positive results allow us to resolve an important and timely question regarding the pathway by which non-visual information reaches the visual cortex of the congenitally blind, and to determine its time course.

## Materials and methods

### Participants

One congenitally blind and two sighted men participated in the study. The sighted men were right-handed, 29 and 33 years of age. The blind man was left-handed and 42 years of age, who became blind immediately after birth due to retinopathy of prematurity. He is fluent in Braille reading, which he uses on a daily basis since the age of six. RIKEN Ethics Committee approved the study and subjects gave written informed consent before the experiment. The congenitally blind subject recruited for this study has previously participated in several PET and fMRI studies conducted with the tongue display unit, which is a sensory substitution device that translates visual information into tactile stimulation that is transmitted to the tongue. Such stimuli strongly activate the occipital cortex (Ptito et al., 2005, 2012; Kupers et al., 2010; Matteau et al., 2010).

### Stimuli

Electrical pulses were delivered transcutaneously to the left and right median nerve at the wrist, using surface electrodes (cathode proximal) connected directly to the photoelectric stimulus isolation unit of a Grass Model S8800 electrical stimulator. A ground band was placed round the forearm above the stimulating electrodes in order to minimize the artifactual magnetic fields caused by the stimulus current. The arms were well-covered to prevent cooling throughout the recording session.

Constant current stimuli with a duration of 0.2 ms at three intensities (Sub, Supra and Motor), were presented with a pseudo-randomized inter-stimulus interval of 600 ± 100 ms. The stimulus intensities were determined based on the metric defined in Ioannides et al. (2002a,b). Briefly, before the experiment, we defined two basic intensity thresholds for both wrists: motor threshold (MTH) as the minimal stimulus intensity producing thumb twitching and sensory threshold (STH) as the minimal stimulus intensity at which the subject was just able to feel a train of stimulus pulses, repeated four times. The STH and MTH for the blind subject were 2.2 and 5.3 mA for the left wrist, and 1.8 and 3.8 mA for right wrist, respectively. The thresholds for the sighted subjects were slightly higher (10–30%), within the ranges found in previous experiments (Ioannides et al., 2002a,b). We then calculated the three experimental stimulus intensities: below sensory threshold, Sub = STH − 0.25 × Δ; above sensory threshold, Supra = STH + 0.25 × Δ; and above motor threshold, sufficient to induce a clear thumb twitching, Motor = MTH + 0.25 × Δ, where Δ = MTH − STH. The adjustment of the stimulus intensities relative to MTH and STH allows precise and objective control over the stimulus effects for each subject. Specifically, the intensities defined in this way provide a clear perceptual differentiation of stimuli, such that Sub stimuli are never perceived, Supra stimuli are clearly perceived, and Motor stimuli produce small, but clear thumb twitch with no pain sensation.

The use of stimuli at different intensities together with the analysis methods described below (see sections Data analysis) allowed us to study the somatosensory processing in the brain maximally independent of general stimulus-unrelated factors, such as subject's arousal and cognitive state, gaze direction, etc. We used the Motor stimuli to elicit strong and clear somatosensory responses, as has been repeatedly done in earlier studies (Ioannides et al., 2002a,b; Papadelis et al., 2009, 2011, 2012), and used the Sub stimuli as the main baseline. However, the contrast of responses to Motor versus Sub stimuli produces widespread brain activations that in addition to the stimulus processing relate also to general arousal and cognitive processes such as awareness and attention. Therefore, in the current study, we used the Supra stimuli as additional baseline to account for such effects.

During the experiment sighted subjects fixated a small cross presented on a screen in front of them. Thus they received visual input, which is missing in the blind subject. Such input is weak, constant, and independent of the somatosensory stimuli; it is also present in all runs. Since our analysis [see section Statistical parametric mapping (SPM)] relies on statistical comparison of responses in different runs, the additional effect of visual input in the sighted subjects will be largely eliminated from the results.

### Recording and preprocessing

MEG signals were collected using a 151-channel whole-head Omega system (CTF/VSM MedTech Ltd.) at a sampling rate of 1250 Hz and hardware band-pass filter of 0–400 Hz. In synchrony with the MEG signals, vertical and horizontal electrooculogram (EOG) and electrocardiogram (ECG) data were also recorded.

We recorded 12 stimulation runs for each participant: two repetitions of each of the three stimulus intensities on each wrist. In each Sub and Supra run, 120 stimuli were delivered, while in the Motor runs, either 80 or 120 stimuli were delivered. Throughout the recording, participants were comfortably seated in a magnetically shielded room and were asked to keep their head still.

The subject's head location relative to MEG sensors was recorded at the beginning and end of each run, using head localization coils attached to the subject's head. Co-registration of the MEG sensors with the individual high-resolution anatomical MRIs was accomplished using a procedure described in Poghosyan and Ioannides (2007), which provides a co-registration accuracy of 1 mm (Hironaga and Ioannides, 2002).

The recorded MEG signals were converted offline to 3rd order synthetic gradient and were band-pass filtered between 1 and 300 Hz, with notches at 50 Hz and its harmonics. One noisy channel (MLT26) was removed. Then the signals were visually inspected for possible artifacts. Independent component analysis in conjunction with vertical EOG and ECG signals was used to remove eye blink and cardiac artifacts, respectively. The processed MEG signals were divided into 700 ms trials, from −200 to 500 ms with respect to stimulus onset.

### Data analysis

#### Source analysis

We performed a source analysis of the MEG signals for each single trial using *standard* magnetic field tomography (MFT) (Ioannides et al., 1990; Taylor et al., 1999; Ioannides, 2006; Poghosyan and Ioannides, 2007, 2008; Ioannides et al., 2012b), with a realistic head model. MFT is a robust and accurate non-linear method that has been successfully used in many prior studies for identifying somatosensory-evoked neural responses. We performed the forward problem computations by means of the symmetric boundary element method (BEM) implemented in OpenMEEG software package (Kybic et al., 2005; Gramfort et al., 2010, 2011). MFT and OpenMEEG are state-of-the-art methods for inverse and forward computations, respectively, that have performed well in tests with computer generated and real data (Poghosyan and Ioannides, 2008; Papadelis et al., 2009; Gramfort et al., 2010).

We applied MFT independently to every timeslice (0.8 ms) of each single trial (from −200 to 500 ms) to estimate the three-dimensional distribution of current density vectors (CDV, current sources) throughout the brain. MFT solutions at each timeslice are described by continuous functions that can be computed at any point in space. However, to raise storage efficiency the current density vectors are saved at representation points only, which make up a 17 × 17 × 17 grid that covers the whole brain with inter-point distance of about 8 mm. This results in time-dependent sequences of whole-brain single-trial CDV maps, i.e., spatio-temporal CDV-maps. High-resolution (1 × 1 × 1 mm) CDV-maps are derived from these representation points by using smooth quintic polynomial interpolation. Tests with computer simulations and a denser grid (inter-point distance of 1 mm) showed that the quintic interpolation estimated the maxima of activity with ~1 mm accuracy.

To study somatosensory-evoked brain responses in the frequency domain, we Fourier transformed each single-trial spatio-*temporal* CDV-map (i.e., the MFT estimate) to a spatio-*frequency* CDV-map with a frequency resolution of 0.2 Hz. To this end, for each single trial, FFT was applied to the time course of the MFT estimated CDV at each source space representation point, component-by-component.

#### Statistical parametric mapping (SPM)

The availability of single trial CDV-maps provides a high statistical power that permits statistical analysis of individual subject data. We used Student's *t*-tests to contrast CDV-maps obtained from runs with different stimulus intensity. The datasets for statistical comparisons were formed by pooling together the moduli of CDV at the same latency or frequency across all trials of the same run. We then used these datasets in two contrasts: Motor vs. Supra, and Motor vs. Supra and Sub pooled together. After each statistical comparison, we moved the latency or frequency forward by one time or frequency point (0.8 ms or 0.2 Hz), varying from −200 to 500 ms with respect to stimulus onset and from 0 to 100 Hz, respectively. In the context of specific contrasts (e.g., Motor vs. Supra), the comparisons were fully combinatorial that is, each run of one intensity was contrasted to each run of the other. Likewise, combining Supra and Sub from two repetitive runs at each intensity produced four distinct datasets (i.e., Supra run1 with Sub run1, Supra run1 with Sub run2, Supra run2 with Sub run1, and Supra run2 with Sub run2).

This procedure produced sets of SPM-maps in time (spatio-temporal SPM-maps) and frequency (spatio-frequency SPM-maps) domains with considerable variability across runs. With two runs for each stimulus intensity, the fully combinatorial Motor vs. Supra comparison produced a set of four SPM-maps in each domain (Motor run1 vs. Supra run1, Motor run1 vs. Supra run2, Motor run2 vs. Supra run1, and Motor run2 vs. Supra run2), while the comparison of Motor vs. Supra/Sub merged produced a set of eight SPM-maps: fully combinatorial merging of two runs of Supra and Sub formed four merged datasets, which were then compared to each of the two Motor runs.

To overcome the variability across runs and focus on the significant brain responses, we combined corresponding SPM-maps of each set to produce activation maps in the time (spatio-temporal activation maps) and frequency (spatio-frequency activation maps) domain. For each contrast, a source space representation point in the combined activation map was considered active if that point showed a significant change of activity in *all* SPM-maps of the set. We applied a Bonferroni corrected statistical threshold of *P* < 0.0001. The maps produced in this way from the two contrasts (i.e., Motor vs. Supra and Motor vs. Supra/Sub) were almost identical. To produce activation maps common across subjects, the individual subject SPM-maps were first transformed to common Talairach space (Talairach and Tournoux, 1988). For display purposes, the resultant activation maps in Talairach space were back-transformed to the source space of one of the subjects and projected on his MRI.

#### Region of interest (ROI) and regional activation curves (RAC)

The averaged CDV-maps and SPM-based activation maps were visually inspected, and we identified brain regions that exhibited distinct activations and consistently dominated the responses in the Motor runs. Within the first 30 ms post-stimulus, we identified regional activations on both banks of the central sulcus in S1 and M1, and bilaterally in the secondary somatosensory cortex (S2). For each subject, the centroids of activations were designated as centers of spherical ROIs with a radius of 0.8 cm. The principle direction for each ROI, which is the dominant orientation of the CDV in that ROI, was calculated using circular statistics (Fisher, 1993), an established framework for identification of statistically significant distributions based on both magnitude and direction of vectors (Ioannides et al., 2005, 2012a,b; Poghosyan and Ioannides, 2007, 2008). Reference to probabilistic cytoarchitectonic maps (Eickhoff et al., 2005, 2006, 2007) confirmed that the dorsal ROIs around the central sulcus were indeed within S1 (most consistently in BA 3b, but also in areas 1, 2, and 3a) and M1 (BA 4a and 4p), while the ventral ROIs were within subdivisions of S2.

We identified a large number of functionally defined ROIs from the averaged CDV- and SPM-based activation maps. For the purpose of network analysis, we reduced the number of ROIs to 14 within each hemisphere, based on their signal-to-noise ratio (SNR, see below).

For every single trial and ROI, we computed a regional activation curve (RAC) which describes the activation time course of a ROI along its principal direction. RACs were generated from single trial spatio-temporal CDV-maps by integrating, for each timeslice of 0.8 ms, the projections of the CDV along the principle direction in the ROI. The RACs provide a convenient tool to study the time course and time-frequency characteristics of each ROI and allow to quantify the functional connectivity between ROIs, and hence to trace the underlying functional networks.

To select the ROIs with a high SNR for the network analysis, we computed their SNR (Raz et al., 1988) from ensembles of single trial RACs, as described in Laskaris and Ioannides (2001) using a 6.4 ms moving window with a step of 0.8 ms (see also Ioannides et al., 2005, 2012b). ROIs with SNR above 0.2 at any time point in the first 100 ms were selected for the network analysis.

#### Time-frequency analysis

Time-frequency analysis was applied to RACs from a selected subset of key ROIs (left and right S1, S2, V1, V5, thalamus, and parietal areas BA 7 and 40). We used Morlet wavelet transform with σ = 6 cycles of sinusoidal oscillation to compute the instantaneous power of single trial RACs.

#### Connectivity analysis using mutual information (MI)

MI is a non-linear measure of relatedness of two discrete random variables based on information theory. Time-delayed MI has been successfully employed in a wide range of applications to estimate information flow between two time series (Alonso et al., 2010; Jin et al., 2010; Albers and Hripcsak, 2012), including to quantify the functional linkage between brain areas (Ioannides et al., 2000, 2002a, 2004a,b). Consider two time series *X* and *Y*, of the same length, but with the second time series sampled with a delay τ relative to the first. Time-delayed MI between these two time series can be expressed in terms of entropy:

I(X, Y; τ)=H(X)+H(Y)−H(X, Y; τ)

where *H*(*X*) and *H*(*Y*) are the marginal entropies of *X* and *Y* respectively, and *H*(*X*, *Y*; τ) is their joint entropy. We use Renyi's entropy to compute MI (Renyi, 1970; Kwapien et al., 1998; Ioannides et al., 2000).

$$H_{\alpha }(X)=\frac{1}{1-\alpha }\text{In}\sum_{i=0}^{n}p_{x}^{\alpha }(i)$$

where *p*_*x*_(*i*) is the probability of instantaneous amplitude *x* falling in the *i*th bin, after splitting the whole range of amplitudes of *X* into *n* bins $\left(\sum_{i=1}^{n}p_{x}\left(i\right)=1\right)$. Note that in the limit α → 1, Renyi entropy converges to standard Shannon entropy. Thus, we used the following formula to compute time-delayed Renyi MI:

$$I_{\alpha }(X,\;Y;\;\tau )=\frac{1}{1-\alpha }\ln\frac{\sum_{i}p_{x}^{\alpha }(i)\sum_{j}p_{y}^{\alpha }(j)}{\sum_{i,\;j}p_{x,\;y}^{\alpha }(i,\;j;\;\tau )}$$

The joint probability *p*_*x*, *y*_(*i*, *j*; τ) is the probability of instantaneous amplitude *x* ∈ *X* falling in the *i*th bin and simultaneously (τ shifted) instantaneous amplitude *y* ∈ *Y* falling in the *j*th bin. Here we used Renyi's entropy of fourth order (α = 4) and split the time series amplitudes into ten bins (*n* = 10). More details about the MI analyses employed here can be found elsewhere (Kwapien et al., 1998; Ioannides et al., 2000).

We computed the MI between each pair of selected ROIs from the averaged RACs of the first Motor run, using a 24 ms moving window. We centered the moving window at latency *t* ms for the first ROI (ROI1) and at *t* + τ for the second ROI (ROI2). The positive (negative) delay τ indicates that ROI1 leads (follows) ROI2. For a fixed pair of ROIs and moving time window the MI then becomes a function of *t* and τ, which we denote as *M*(*t*, τ). The MI value, *M*(*t*, τ), identifies how the activity of ROI1 in the 24 ms window, centered at *t*, is linked to the activity in a similar 24 ms window of ROI2, centered at *t* + τ latency. We computed the MI for all (*t*, τ) pairs, where −200 ≤ *t* ≤ 500 ms, and −100 ≤ τ ≤ 200 ms, building a map of MI in the (*t*, τ) plane for each pair of selected ROIs. It is important to realize that this linkage does not necessarily imply a causal influence. The link identified by a high MI value may be due to common interaction with a third area via links with different time delays.

We used the MI analysis to identify how activity elicited by median nerve stimulation reaches the visual cortex. In particular, our goal was to identify the fastest pathways. Thereto, we considered the linked activities from −50 to 100 ms with respect to stimulus onset and only relatively short delays, 5 ≤ |τ| ≤ 20 ms. Assuming that axonal conduction time plays an important role in the observed MI values, the restriction on the delay values (5 ≤ |τ| ≤ 20) has two effects. First, eliminating the delays close to zero lag removes common input from a third area and apparent connectivity due to an overall change in brain activity that is spatially non-specific. Second, restricting the delays to 20 ms maximally allows only interchanges (between fairly distant areas) via one or at most a few synapses. The MI maps for the two sighted subjects were similar so they were combined when appropriate to reduce the number of figures. For this combination we first normalized *M*(*t*, τ), values for each subject and ROI pair and then used a common threshold *c* to obtain the average MI map as follows: the average *M*(*t*, τ) was set to zero if the normalized MI value was less than *c* for any subject. For each remaining pair, the average MI was set as the average of the normalized MIs across the two subjects. We used a threshold value of *c* = 0.4.

We pooled the results of individual MI and/or average MI maps as influence diagrams using a procedure described earlier (Ioannides et al., 2004a). Briefly, these diagrams arrange ROIs in successive rows and time flows from left to right. Arrows are used to define the linkage between ROIs (read directly from the MI maps). Influence diagrams derived from MI maps summarize in one display large amounts of information regarding functional linkage between many ROIs.

## Results

In order to characterize real-time brain responses that are precisely time-locked to the stimulus, we relied on high-resolution spatio-temporal activation maps. To study weaker responses that are not precisely time-locked to the stimulus onset, we relied on spatio-frequency activation maps derived from the entire trial period, i.e., from −200 to 500 ms. On the basis of these results, we computed functional connectivity measures that traced the pathways involved in visual cortex activation following somatosensory stimulation.

### Brain responses around the central sulcus

#### Averaged spatio-temporal CDV-maps

**Figure 1:**
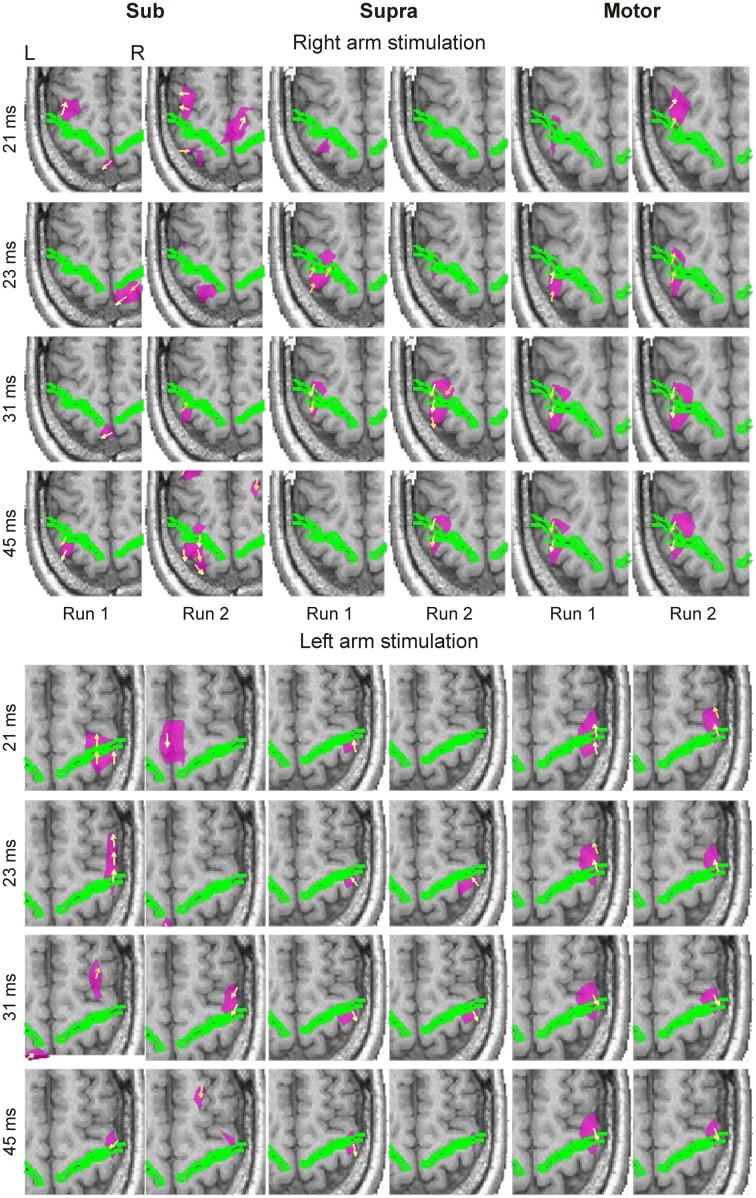
**Averaged spatio-temporal CDV-maps of the blind subject are shown for both runs of Sub (columns 1 and 2), Supra (columns 3 and 4) and Motor (columns 5 and 6) intensities at four latencies (indicated at the left side of each row) in response to right (upper block) and left (lower block) arm stimulations**. The axial slice of MRI best covering the activated part of central sulcus, indicated by a green outline, is shown. Yellow arrows show the direction of CDV. Pink contours encompass regions where the normalized magnitude of CDV is above 0.9. For display purposes the CDV magnitudes were normalized independently for each image. This made all the activations clearly visible. L, left; R, right.

We identified reproducible brain responses on both banks of the central sulcus within 50 ms of the stimulus onset from the averaged spatio-temporal CDV-maps in all subjects (Figure 1). In response to Motor and Supra stimulation, these responses were clearly localized in contralateral S1 (BA 3b) and M1 (BA 4a), and were evident as early as 20 ms post-stimulus. The responses were very similar in sighted and blind subjects. Responses to Sub stimuli were weaker and labile, but we identified activations at the expected locations around the contralateral central sulcus, especially after 40 ms post-stimulus. In general, the strength and reproducibility of the evoked responses decreased with stimulation intensity.

The first response between 18 and 23 ms, corresponding to the electroencephalography (EEG) N20 component, was very precise in terms of its localization and peak latency. The response to Supra stimuli occurred initially in the posterior bank of the central sulcus, and then by 23 ms crossed to the motor side on the anterior bank. This shift occurred even earlier in response to Motor stimulation.

#### Temporal pattern of regional responses

**Figure 2:**
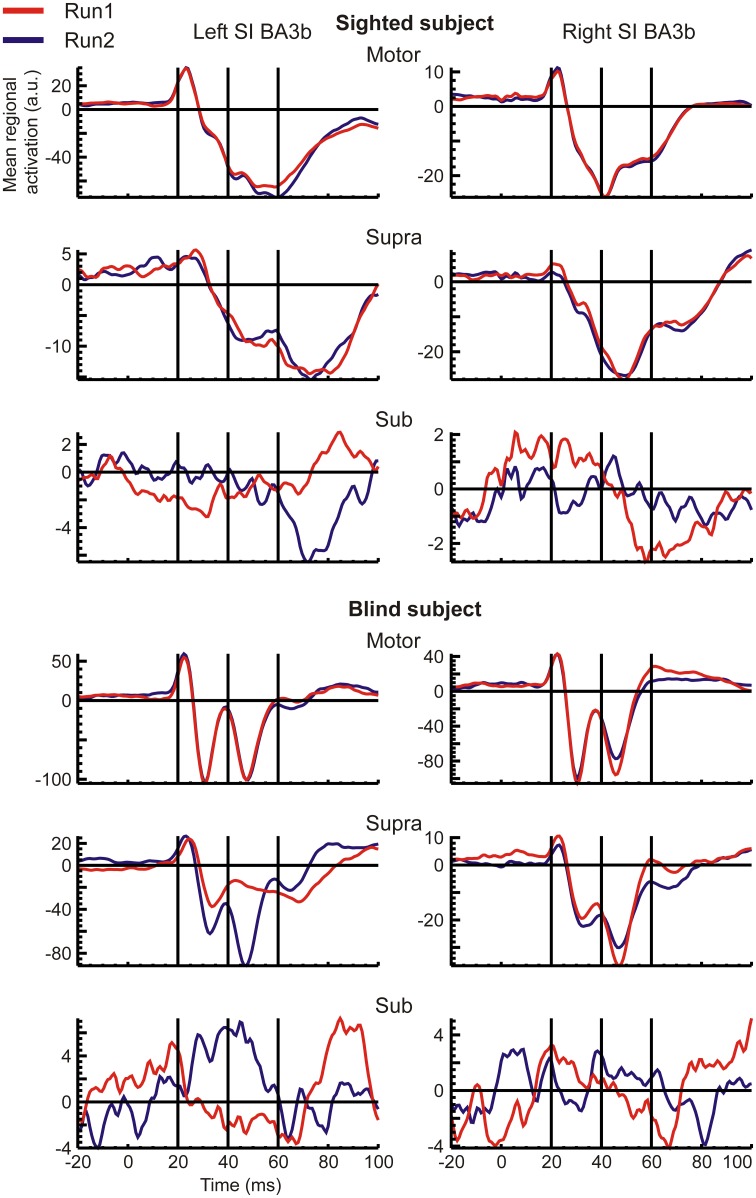
**Mean regional activation curves (RAC) of left (left column) and right (right column) S1 ROIs, generated in response to contralateral stimuli are shown for both runs (overplotted red and blue curves) of each stimulus intensity (indicated on top of each rows) in a sighted (upper half) and the blind (lower half) subjects**.

**Figure 3:**
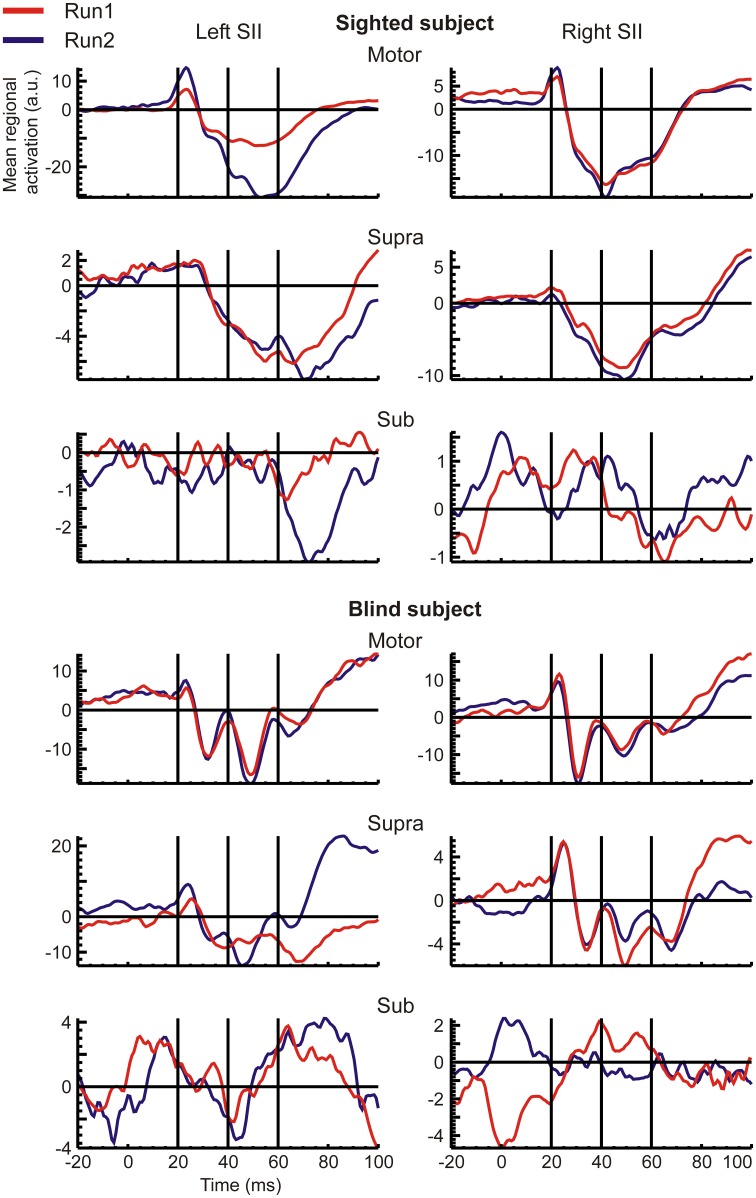
**The same as the Figure 2, but for the S2 ROIs**.

RACs generated from the S1 and S2 ROIs revealed different temporal response patterns across subjects after the initial peak at 20 ms (Figures 2, 3), even for the strong and reproducible S1 responses (Figure 2). Despite this variability, there was a remarkable similarity between response patterns in the left and right hemisphere ROIs of the same subject. In accordance with evidence from spatio-temporal CDV-maps (Figure 1), analysis of RACs revealed a very high reproducibility of brain regional responses to Motor stimuli and to a lesser extent also to Supra stimuli. Sub stimulation evoked no clear responses. The overall magnitude of brain activations was about twice stronger in S1 (Figure 2) than in S2 (Figure 3).

#### SPM-based grand activation maps

**Figure 4:**
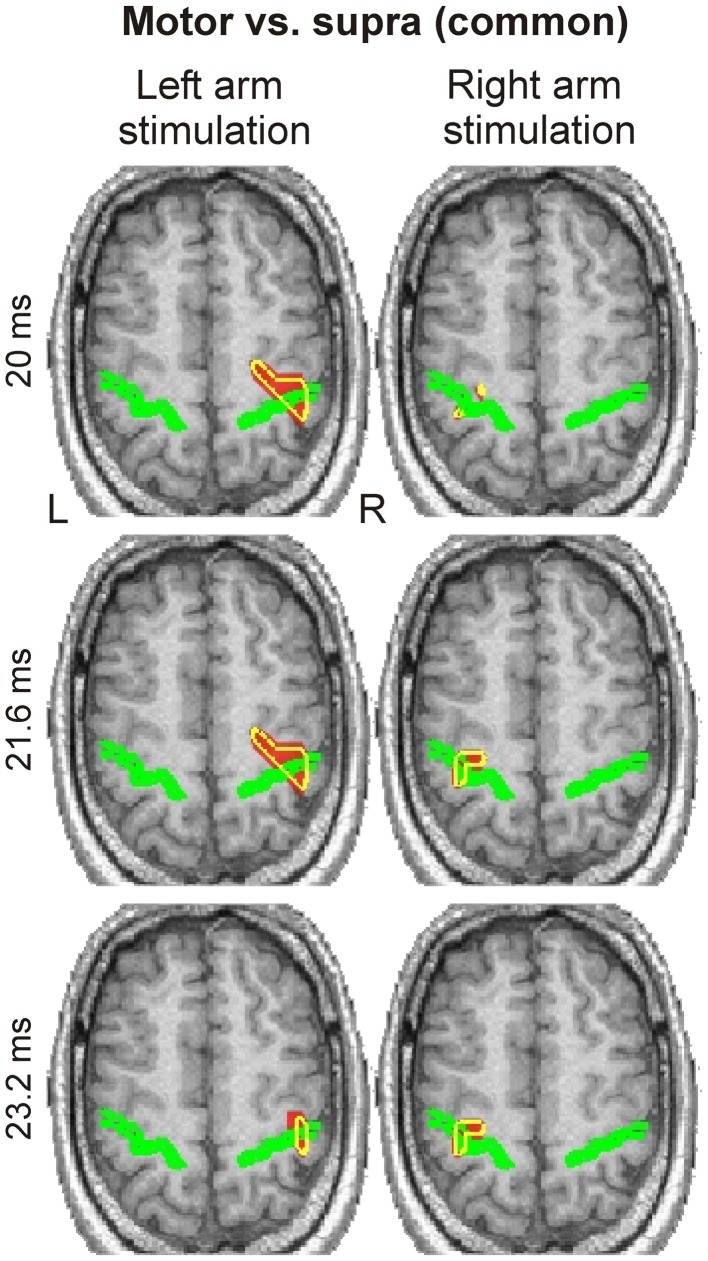
**Spatio-temporal grand activation maps derived from “Motor vs. Supra” contrast are shown for left (left column) and right (right column) arm stimulation at three latencies (indicated at the left side of each row)**. The common across all three subjects activations are projected on the MRI of the blind subject. The axial slice of MRI best covering the central sulcus, indicated by a green outline, is shown. Yellow contours encompass regions with statistically significant (*P* < 0.0001) increases in activity in all three subjects.

Spatio-temporal grand activation maps (common in all three subjects) derived from the “Motor vs. Supra” contrast revealed responses only around the contralateral central sulcus, namely in S1 and M1, at latencies between 20 to 23.2 ms (Figure 4). No other activation was evident in these maps.

**Figure 5:**
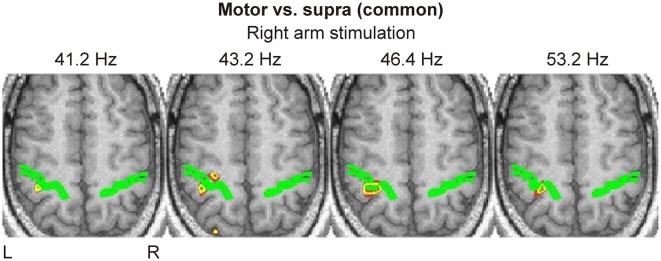
**Spatio-frequency grand activation maps derived from “Motor vs. Supra” contrast are shown for right arm stimulation at four frequencies (indicated on top of each image)**. The same conventions as in Figure 4 are used.

Activations within the same brain regions were also evident in the spatio-frequency grand activation maps for right arm stimulation (Figure 5); these activations were precisely focal and restricted to a narrow range of frequencies in the low gamma band, from 41.2 to 53.2 Hz. For much of these frequencies, we identified the response on the posterior bank of the central sulcus (S1). We found focal activation in the anterior bank and in the posterior parietal cortex at only at one frequency (43.2 Hz).

### Brain responses in the visual areas

Visual inspection of averaged and single-trial spatio-temporal CDV-maps revealed weak and intermittent activations in occipital cortex, which were not precisely time-locked to the stimuli, but occurred consistently in the same brain areas. We focused on the SPM-based spatio-frequency activation maps derived from the contrast “Motor vs. Supra/Sub.” The availability of single-trial CDV-maps provided a high statistical power for identifying weak somatosensory-evoked responses in the visual cortex, while the analysis of CDV-maps in the frequency domain allowed us to explore time-unlocked neural responses.

**Figure 6:**
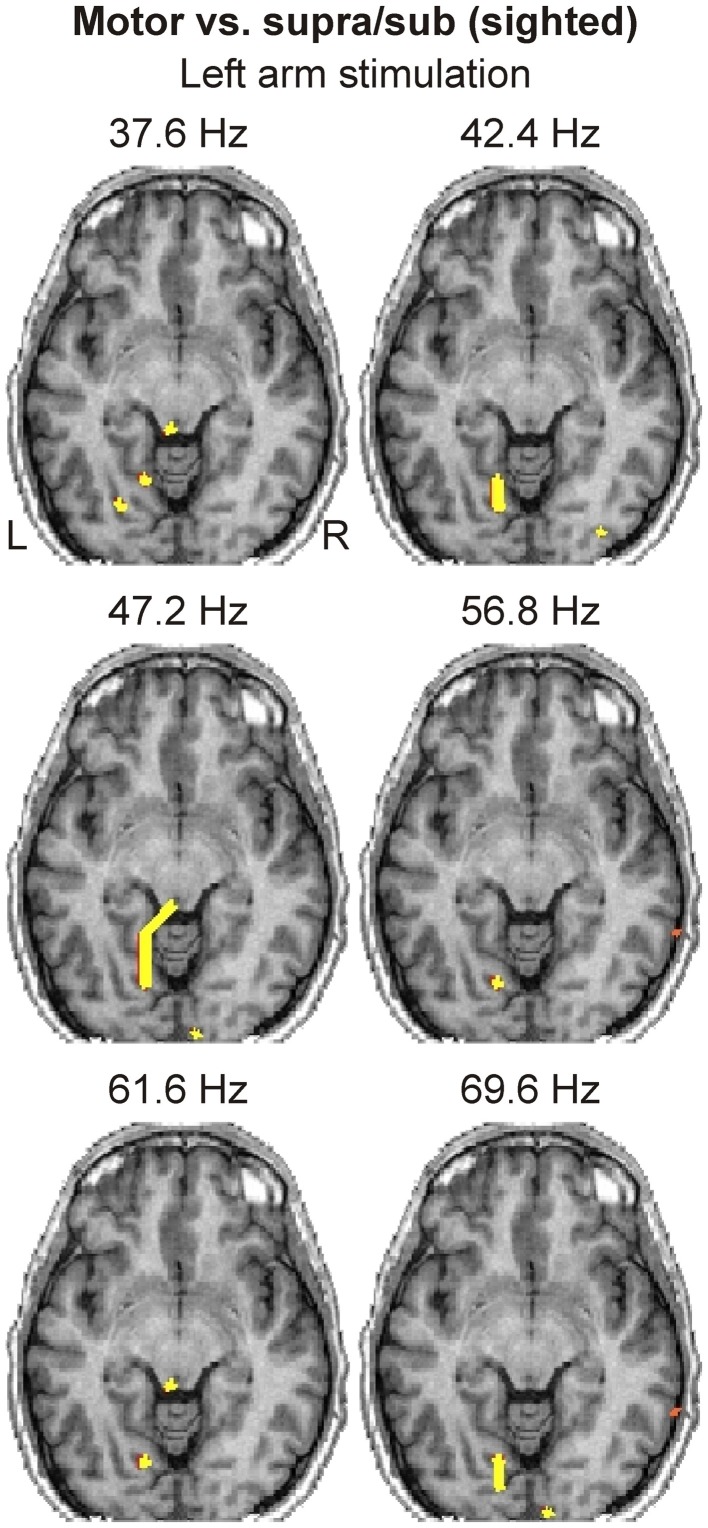
**Spatio-frequency activation maps of a sighted subject derived from “Motor vs. Supra/Sub” contrast are shown for left arm stimulation at six frequencies (indicated on top of each image)**. The axial slice of MRI best covering the visual cortex activations is shown. Yellow contours encompass regions with statistically significant (*P* < 0.0001) increases in activity.

We identified occipital activations in all subjects. The two sighted subjects showed consistent focal γ-band responses only in response to left arm stimulation in the ipsilateral (left) extrastriate visual areas (Figure 6). We identified less consistent responses for right arm stimulation and in other frequency bands. In general, sighted subjects only showed some focal activations, mostly in the ipsilateral visual cortex.

**Figure 7:**
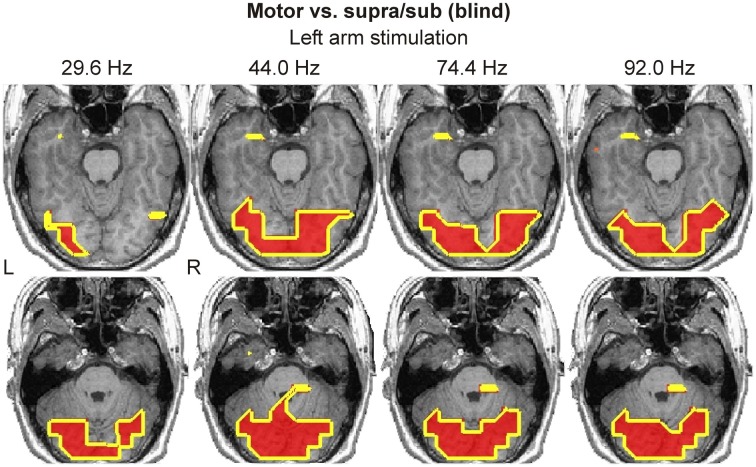
**Spatio-frequency activation maps of the blind subject derived from “Motor vs. Supra/Sub” contrast are shown for left arm stimulation at four frequencies (indicated on top of each column)**. Two axial slices of MRI best covering the visual cortex activations are shown. Yellow contours encompass regions with statistically significant (*P* < 0.0001) increases in activity.

In contrast, the spatio-frequency activation maps in the blind subject, based on the same contrast, revealed widespread activations in bilateral ventral visual cortex (Figure 7). Such activations were present starting from α-band to 100 Hz. We observed a striking difference between the responses in these occipital ventral areas in terms of spatial extent and range of frequencies in favor of the blind subject who exhibited a much higher activity.

### Time-frequency analysis

**Figure 8:**
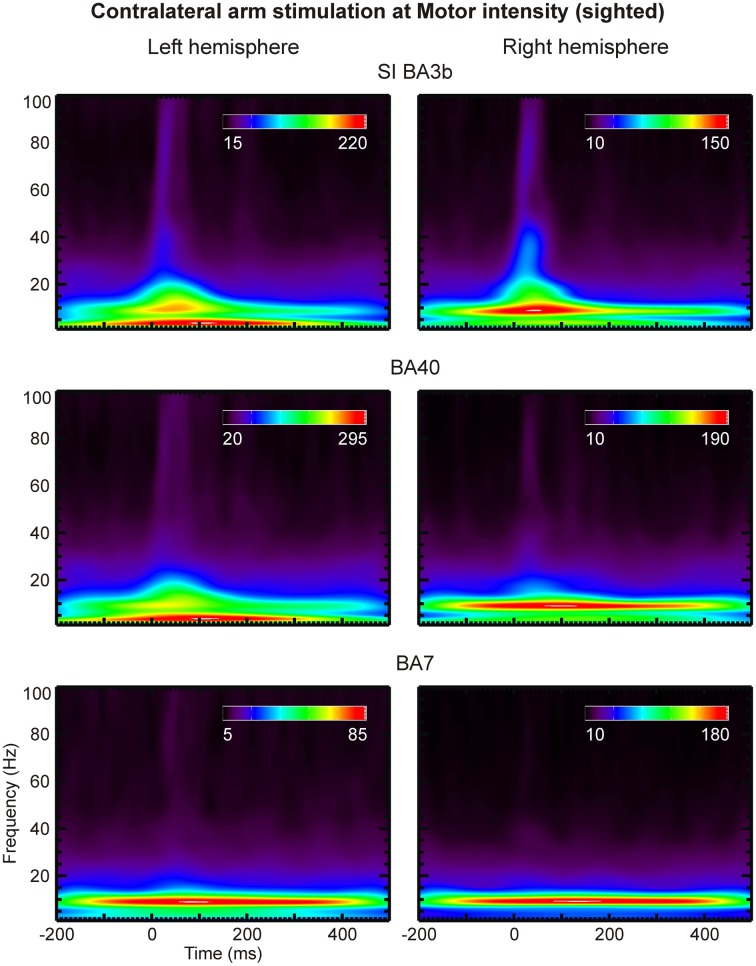
**Scalograms of three left (left column) and right (right column) hemisphere ROIs (indicated on top of each row) are shown for contralateral arm stimulation at Motor threshold in a sighted subject**. The coefficient values are given in arbitrary units.

**Figure 9:**
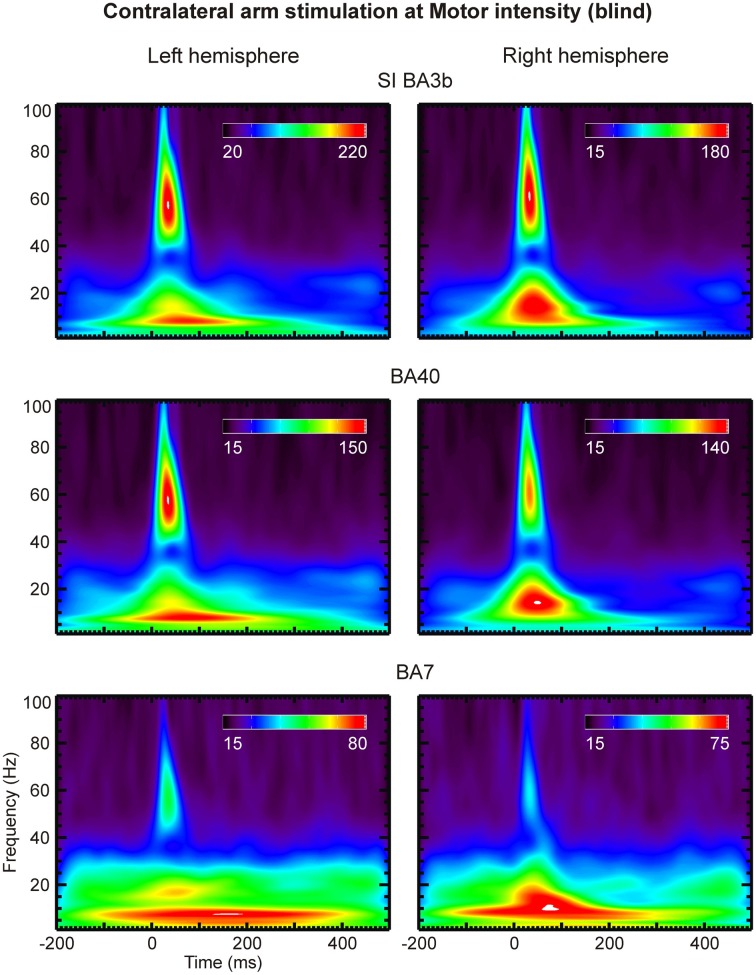
**The same as the Figure 8, but for the blind subject**.

Analysis of the CDV and SPM-based activation maps in the time and frequency domains identified a large set of ROIs. We selected a subset of key ROIs (left and right S1, S2, V1, V5, thalamus, and parietal areas BA 7 and 40), thought to be involved in the putative pathway for relaying somatosensory information to the visual cortex, and submitted them to more refined time-frequency analysis by means of Morlet wavelet transform (Figures 8, 9).

Compared to lower frequencies, γ-band responses were substantially more pronounced in the blind than the sighted subjects. No notable difference was evident between the scalograms of sighted subjects. We observed strong γ-band oscillations in areas S1 (BA 3b), BA40 and BA7 of the blind subject at latencies of 20–50 ms post-stimulus and at frequencies of 45–70 Hz (Figures 8, 9). These oscillations were present in response to all stimulus intensities, including Sub stimulation, but their magnitude decreased as stimulus intensity decreased.

### Connectivity analysis using MI

**Figure 10:**
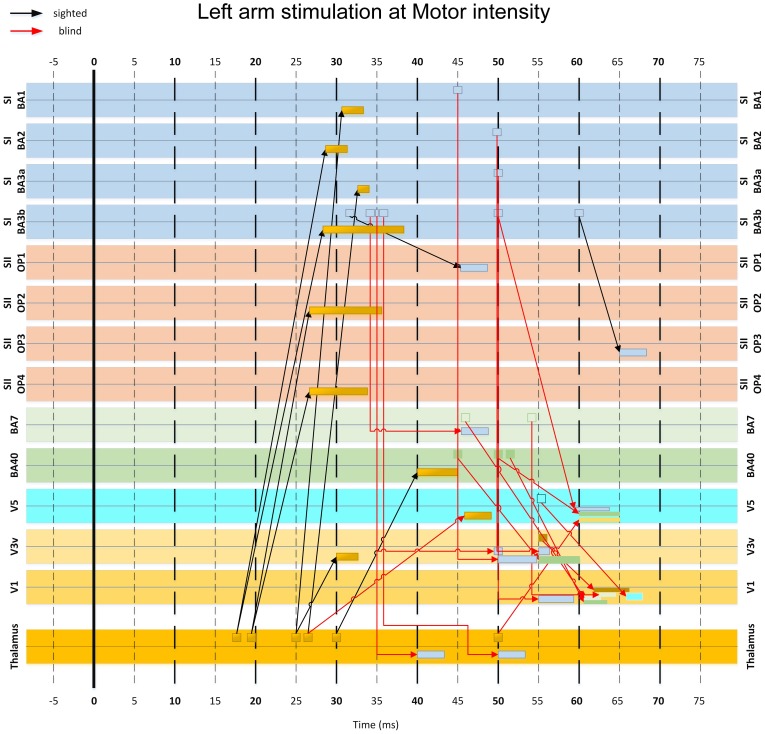
**MI-based influence diagram derived from left arm stimulation at Motor intensity. Interregional connection among 14 selected ROIs (indicated at the sides of each row) are shown for sighted (black arrows with MI value threshold *c* > 0.4) and the blind (red arrows with MI value threshold *c* > 0.9) subjects**. Arrows indicate the direction of the links in terms of corresponding response latencies and do not necessarily imply causal influence. The base of the arrow indicates the onset latency of the first linked activation. The rectangle pointed by the arrowhead indicates the entire duration of the second linked activation.

**Figure 11:**
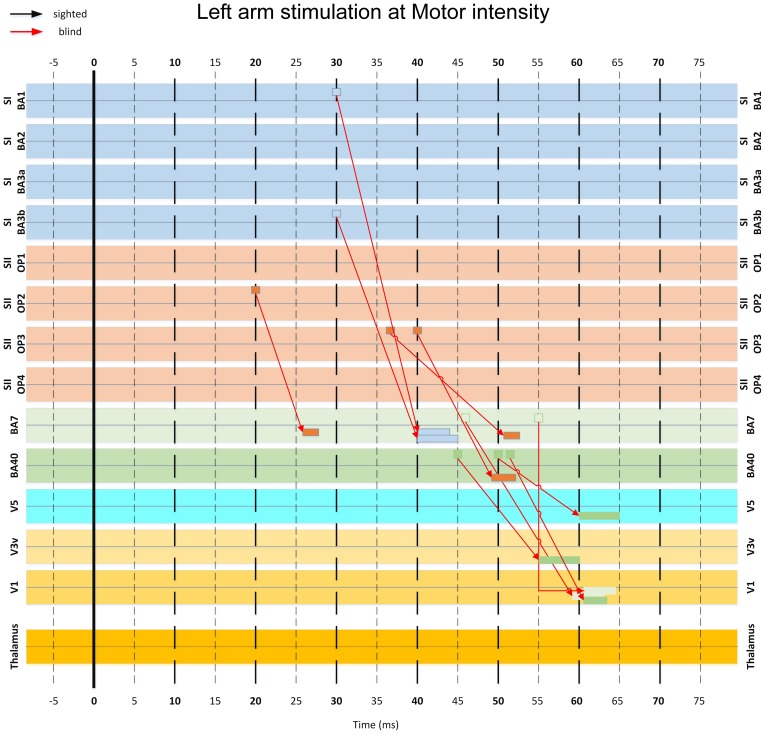
**A more detailed version of part of the diagram in Figure 10, showing the key connections involved in the pathway from the somatosensory to the visual cortex via posterior parietal areas**. Note that some connections appearing in this figure have been omitted in Figure 10 for clarity.

We analyzed the MI-based functional connectivity between a selected subset of ROIs in an effort to identify the neural pathways conveying somatosensory inputs to the visual cortex. This analysis revealed some important differences between the blind and sighted subjects (Figures 10, 11). The initial activity in the thalamus between 15 to 30 ms post-stimulus was connected to activities in the somatosensory (S1 and S2) and posterior parietal (areas BA 40 and 7) cortices, at 25–35 ms and 40–50 ms, respectively. At the high threshold used to select the interregional links, these thalamo-cortical connections were clearly identified in sighted subjects (Figure 10), whereas in the blind subject they were evident only at a lower threshold.

In the blind but not in the sighted subjects, activity from somatosensory cortex at 30–40 ms converged to conduit parietal areas BA40 and BA7 at 40–55 ms. An earlier isolated connection from S2 at 20 ms to BA7 at 27 ms was also present (Figure 11).

Finally, the responses from parietal areas and somatosensory cortices converged to visual cortical areas V1, V3, and V5 from 50–65 ms (Figures 10, 11). The linked activity in the visual cortex was first evident in V3 starting from 50 ms, and then in V5 and V1, with activity occurring in V5 few ms before V1. In sighted subjects, no linked activity was found in the first 100 ms of post-stimulus period that led from the somatosensory to the visual cortex through any of the studied ROIs.

## Discussion

### Early somatosensory evoked responses around the central sulcus

We identified highly reproducible somatosensory evoked responses at ~20 ms post-stimulus on both banks of the central sulcus in all subjects. These responses were very similar in sighted and blind subjects. Their anatomical locations and timing agree well with findings from monkey (Pons et al., 1992) and human studies, using intracranial recordings (Balzamo et al., 2004) and MEG (Inui et al., 2004). In general, the spatio-temporal pattern of responses in the first 100 ms of the post-stimulus period in sighted subjects were within the range of response patterns found in our previous studies with similar stimuli and subjects 24–50 years of age (Ioannides et al., 2002a,b).

Statistical analysis in the time domain revealed early (~20 ms) focal responses in S1 (BA 3b) and M1 (BA 4a) in all subjects, which were clearly evident in the SPM-based grand activation maps. The same analysis in the frequency domain identified responses at precisely the same locations in the γ-band (41–53 Hz), attesting the significance of this frequency band in the generation of early sensory-evoked responses.

The fine localization and time course of early responses in S1 and S2 were highly reproducible across runs. Moreover, we found a remarkable similarity between the activation time courses of the prominent homologous areas in the left and right hemispheres of each subject. In contrast, the time courses had a distinct morphology that was different from subject to subject, with only the first post-stimulus response in S1 at ~20 ms consistent across subjects. These results suggest that the early processing follows the same route from the thalamus to the somatosensory and motor cortical areas with the same timing in both hemispheres of all subjects.

The early averaged responses clearly differed in magnitude between the blind and sighted subjects. In the blind subject, S1 and S2 responded about twice as strongly as in the sighted subjects, in agreement with recent results (Dayananda et al., 2008). Moreover, the high γ-band (>60 Hz) activations in the sensorimotor cortex were stronger and more consistent in the blind subject.

The time-frequency analysis showed that the γ-band responses in selected ROIs were well-organized in time. Compared to the lower frequency responses, they were substantially more pronounced in the blind subject. We identified clear γ-band (45–70 Hz) responses at 20–50 ms post-stimulus in areas around the central sulcus and in the conduit areas in the posterior parietal cortex, BA7 and BA40. The same analysis revealed that the γ-band activity in the visual cortex is not well-organized in time.

### Somatosensory evoked responses in visual cortex

In agreement with our main hypothesis, we identified clear activations in the visual cortex of the blind subject following simple somatosensory stimulation. These activations were present from α-band up to 100 Hz, and they were stronger and more widespread in the γ-band (low and high frequency ranges). Interestingly, we also identified activations in the visual cortex of the sighted subjects, present only in the γ-band. These activations were more focal, and more prominent in the ipsilateral hemisphere. Importantly, the time-frequency analysis did not identify clear γ-band responses in visual cortical areas of blind or sighted subjects, indicating that these are not well-organized in time. Some earlier studies have reported visual cortex activation in sighted subjects in demanding haptic discrimination tasks (Zangaladze et al., 1999; Pietrini et al., 2004; Matteau et al., 2010; Ptito et al., 2012). However, to the best of our knowledge, this is the first report of such activation with simple somatosensory stimuli.

### A pathway for conveying tactile inputs to the visual cortex

We used time-delayed MI analysis to trace the early feedforward wave of activity from the thalamus and somatosensory cortex to the visual cortex. First, this analysis provided evidence of an expected early connection from the thalamus to subdivisions of S1 and S2. In the blind subject only, we traced linked activations from somatosensory to visual cortex through the posterior parietal areas. The processing along this pathway occurred in three stages, characterized by convergence of activities into specific cortical regions. In the earliest stage (15–35 ms), activity from the thalamus proceeded to S1 and S2. In the second stage (40–50 ms), activity passed from S1, S2 and thalamus to BAs 40 and 7 in the posterior parietal cortex. In the final stage (50–65 ms), the activity progressed to visual cortex, first converging in V3, then in V5, and few ms later in V1. This result is in line with earlier results from our laboratory, showing evoked responses to tactile stimuli in occipital areas after 48–60 ms in congenitally blind subjects (Kupers et al., 2006). We did not identify a pathway from somatosensory to visual areas in the sighted subjects.

The feedforward pathway that linked the somatosensory and visual cortices through posterior parietal BAs 7 and 40 in the blind subject agrees with findings from earlier studies using more complex tasks (Buchel et al., 1998; Ptito et al., 2005; Kupers et al., 2010). Activations in similar posterior parietal areas were identified in blindfolded sighted subjects after prolonged training (Merabet et al., 2008) and with induced conflict between intentions and senses (Fink et al., 1999). The short response latencies of these areas are also consistent with recent MEG studies showing that the early (<100 ms) responses in posterior parietal areas are strongly affected by unexpected visual feedback (Wasaka and Kakigi, 2012). The results of the present MI analysis also agree with the prediction of Negyessy et al. (2006) of an integrative role of area V5 and V3 in shaping V1 activity during tactile tasks.

### Identification of subcortical activations

In addition to a number of cortical ROIs, we have identified an ROI in the thalamus of each hemisphere for every subject. We used the contralateral thalamus to the side of stimulation in our MI analysis. The latency of the thalamic response and its connection with the somatosensory cortex identified here agrees well with the findings from the previous literature (Kimura et al., 2008; Papadelis et al., 2012).

In general, thalamic responses are the most difficult to detect and localize using MEG, because of their deep location (close to the center of the skull and far from sensors) and “closed-field” neuronal architecture (contains mainly stellate cells). However, many recent MEG studies have reported activations generated in deep subcortical structures such as thalamus (Attal et al., 2012; Wang et al., 2012; Tenney et al., 2013), including in response to somatosensory stimulation (Hashimoto et al., 2001; Tecchio et al., 2005; Jaros et al., 2008; Kimura et al., 2008; Milde et al., 2009; Papadelis et al., 2012). These results indicate that neural activity in thalamus and other subcortical structures generate detectable MEG signals and can be identified if appropriate methods are used. One of the first attempts to localize thalamic response from MEG signals was done using MFT (Ribary et al., 1991), the inverse method which was used in the current study. Since then MFT methodology and MEG systems have been continually advanced. In the past MFT was successfully used to localize subcortical sources in amygdala, cerebellum and brainstem (Ioannides et al., 2004a,b, 2005; Ioannides and Fenwick, 2005). Moreover, tests using realistically shaped phantom have shown that this methodology can identify thalamic sources with a good accuracy (Papadelis et al., 2009). The current study is the first application of MFT with realistic head model, which further improves the estimates of deep sources such as the thalamic responses.

### Limitations of the study and outlook

Although the study was carried out in a single congenitally blind individual, it yields important results. In the present study, we demonstrated indeed that a simple non-visual stimulus elicits responses in the visual cortex in a congenitally blind participant who has consistently demonstrated with other methods activation of the visual cortex in response to a variety of non-visual stimuli (Ptito et al., 2005, 2012; Kupers and Ptito, 2011). We identified a neural pathway that conveyed somatosensory inputs to the occipital visual areas via posterior parietal cortex and showed that this parieto-occipital pathway is not activated in sighted subjects. Moreover, our results support the claim that it is possible to trace the evolution of activity from sub-cortical areas (Kimura et al., 2008; Papadelis et al., 2012) through sub-divisions of S1, M1 (Inui et al., 2004; Papadelis et al., 2011), S2 and posterior parietal areas.

### Conflict of interest statement

The authors declare that the research was conducted in the absence of any commercial or financial relationships that could be construed as a potential conflict of interest.
